# Baseline EEG-correlates of responders/non-responders to combined antidepressive treatment including transcranial magnetic stimulation

**DOI:** 10.1192/j.eurpsy.2021.308

**Published:** 2021-08-13

**Authors:** E. Iznak, A. Iznak, I. Oleichik

**Affiliations:** 1 Laboratory Of Neurophysiology, Mental Health Research Centre, Moscow, Russian Federation; 2 Clinical Department Of Endogenous Mental Disorders And Affective States, Mental Health Research Centre, Moscow, Russian Federation

**Keywords:** transcranial magnetic stimulation, baseline EEG, prediction of treatment effects, pharmaco-resistant depression

## Abstract

**Introduction:**

Use of combined antidepressive treatment included high-frequency rhythmic transcranial magnetic stimulation (rTMS) of the left dorsolateral prefrontal cortex (DLPFC) is one of the ways for overcoming of pharmaco-resistance in depressive patients.

**Objectives:**

The aim of the study was the search for possible EEG predictors of antidepressive effects of rTMS of the left DLPFC in combined treatment of depression.

**Methods:**

30 female in-patients (F31.3, F33.0, F33.1, by ICD-10; 20-50 years, mean age 36.9±10.3) with pharmaco-resistant depression were enrolled in the study. Treatment included antidepressants (mainly SSRI) and a 3-week course of rTMS (20 Hz) of the left DLPFC. Correlations between pre-treatment EEG spectral power values, and post-treatment quantitative clinical assessments of patients were analyzed. Responders/non-responders were determined by standard criteria of 50% decrease in HDRS-17 scale total scores after treatment course.

**Results:**

Responders (23 out of 30) revealed significant (p<0.05) negative correlations between post-treatment HDRS-17 scores and pre-treatment EEG spectral power in theta-2 (6-8 Hz) and alpha-1 (8-9 Hz) frequency sub-bands in the parietal-occipital-posterior temporal leads. Non-responders (7 out of 30) showed negative correlations between the post-treatment HDRS-17 scores and pre-treatment theta-2 EEG spectral power in the frontal-central-temporal regions of the right hemisphere.

**Conclusions:**

Even brief course of rTMS of the left DLPFC enhances the action of antidepressants, and allows overcoming partially the pharmaco-resistance in depressive patients. Baseline values of theta-2 and alpha-1 EEG spectral power may serve as possible predictors of the effects of combined antidepressive therapy including rTMS. The study supported by RBRF grant No.18-01-00029a.

**Disclosure:**

No significant relationships.

